# Handbook of Materia Medica, Pharmacy, and Therapeutics

**Published:** 1890-06

**Authors:** 


**Handbook of Materia Medica, Pharmacy and Therapeutics.**—Including the Physiological Action of Drugs, the special Therapeutics of Disease, Official and Extemporaneous Pharmacy and Minute Directions for Prescription writing. By SAML. O. L. POTTER, M. A., M. D., Professor of the Theory and Practice of Medicine in the Cooper Medical College of San Francisco; Late A. A. Surgeon U. S. Army. Second edition, revised and enlarged. Pp. xii.-766. Philadelphia: P. Blakiston, Son & Co., 1012 Walnut street. 1890. Price, cloth, $4.00; leather, $5.00.

When the first edition of Dr. Potter's book appeared, now something over three years ago, we spoke in its praise. The profession of

America has now spoken more emphatically than we, by demanding a second edition in so short a time. It has been enlarged and improved, the index alone comprising thirty-seven pages. The titles are conveniently arranged, and the thumb index adds to the comfort of the consultant of its pages.

The subjects treated of in this work are those that every physician must refer to, and it is desirable that the latest and best authority should always be at hand. Whoever purchases Dr. Potter's book will be sure to obtain satisfaction in the direction above named. The type is clear, the paper excellent, and it is one of the prettiest volumes that the famous publishers have lately issued.

